# New insights on the potential anti‐epileptic effect of metformin: Mechanistic pathway

**DOI:** 10.1111/jcmm.17965

**Published:** 2023-09-22

**Authors:** Saud A. Alnaaim, Hayder M. Al‐kuraishy, Ali I. Al‐Gareeb, Naif H. Ali, Athanasios Alexiou, Marios Papadakis, Hebatallah M. Saad, Gaber El‐Saber Batiha

**Affiliations:** ^1^ Clinical Neurosciences Department, College of Medicine King Faisal University Hofuf Saudi Arabia; ^2^ Department of Clinical Pharmacology and Medicine, College of Medicine ALmustansiriyia University Baghdad Iraq; ^3^ Department of Internal Medicine, Medical College Najran University Najran Saudi Arabia; ^4^ Department of Science and Engineering Novel Global Community Educational Foundation Hebersham New South Wales Australia; ^5^ AFNP Med Wien Austria; ^6^ Department of Surgery II University Hospital Witten‐Herdecke, University of Witten‐Herdecke Wuppertal Germany; ^7^ Department of Pathology, Faculty of Veterinary Medicine Matrouh University Matrouh Egypt; ^8^ Department of Pharmacology and Therapeutics, Faculty of Veterinary Medicine Damanhour University Damanhour Egypt

**Keywords:** epilepsy, metformin, seizure

## Abstract

Epilepsy is a chronic neurological disease characterized by recurrent seizures. Epilepsy is observed as a well‐controlled disease by anti‐epileptic agents (AEAs) in about 69%. However, 30%–40% of epileptic patients fail to respond to conventional AEAs leading to an increase in the risk of brain structural injury and mortality. Therefore, adding some FDA‐approved drugs that have an anti‐seizure activity to the anti‐epileptic regimen is logical. The anti‐diabetic agent metformin has anti‐seizure activity. Nevertheless, the underlying mechanism of the anti‐seizure activity of metformin was not entirely clarified. Henceforward, the objective of this review was to exemplify the mechanistic role of metformin in epilepsy. Metformin has anti‐seizure activity by triggering adenosine monophosphate‐activated protein kinase (AMPK) signalling and inhibiting the mechanistic target of rapamycin (mTOR) pathways which are dysregulated in epilepsy. In addition, metformin improves the expression of brain‐derived neurotrophic factor (BDNF) which has a neuroprotective effect. Hence, metformin via induction of BDNF can reduce seizure progression and severity. Consequently, increasing neuronal progranulin by metformin may explain the anti‐seizure mechanism of metformin. Also, metformin reduces α‐synuclein and increases protein phosphatase 2A (PPA2) with modulation of neuroinflammation. In conclusion, metformin might be an adjuvant with AEAs in the management of refractory epilepsy. Preclinical and clinical studies are warranted in this regard.

## 1 INTRODUCTION

Epilepsy is a chronic neurological disease characterized by recurrent seizure which is hypersynchronous neuronal discharge from a specific brain region.^1^ One attack of seizure can occur in any subject that is not regarded as epilepsy, but investigations are warranted to define the underlying cause of the seizure.^2^ However, happening of two or more seizure is defined as epilepsy.^3^ Single seizure warrants a specific definition as it will not occur again or could be the first sign of refractory epilepsy.^3^ Besides, seizure should be differentiated from convulsion which is a term that describes uncontrolled muscle contractions and can be caused by other metabolic disorders such as hypoglycaemia and hypocalcaemia.^4^ However, both seizure and convulsion are often used interchangeably, though epileptic seizures may occur without convulsion.^4^

Epilepsy was recorded throughout ancient history as a spiritual status. The epileptic seizure was mentioned in the Akkadian language in ancient Mesopotamia in 2000 BC.^5^ As well, the epileptic seizure was listed in Hammurabi Code 1790 BC.^5^ In ancient Greece, epilepsy was regarded as a form of spiritual disease so‐called sacred disease.^5^ However, in the fifth century BC, Hippocrates discarded this idea, and epilepsy was caused by a treatable brain disorder.^6^ In the nineteenth century, Jean‐Martin Charcot revealed that epilepsy was misdiagnosed with chronic syphilis and mental disorders.^7^ Phenobarbital in 1912 and phenytoin in 1938 were introduced in the management of epilepsy.^8^

Regarding the prevalence of epilepsy, it affects about 1% of the general population globally till 2020.^9^, ^10^ It has been shown that 80% of epileptic cases worldwide are in developing countries, and it is more common in the elderly.^11^ However, in developed countries, the incidence of epilepsy is more common at extreme ages both in children and the elderly.^12^ Approximately, 5%–10% of old people have a seizure at the age of 80, increasing the chance of a second seizure by 40%–50%.^13^

**FIGURE 1:**
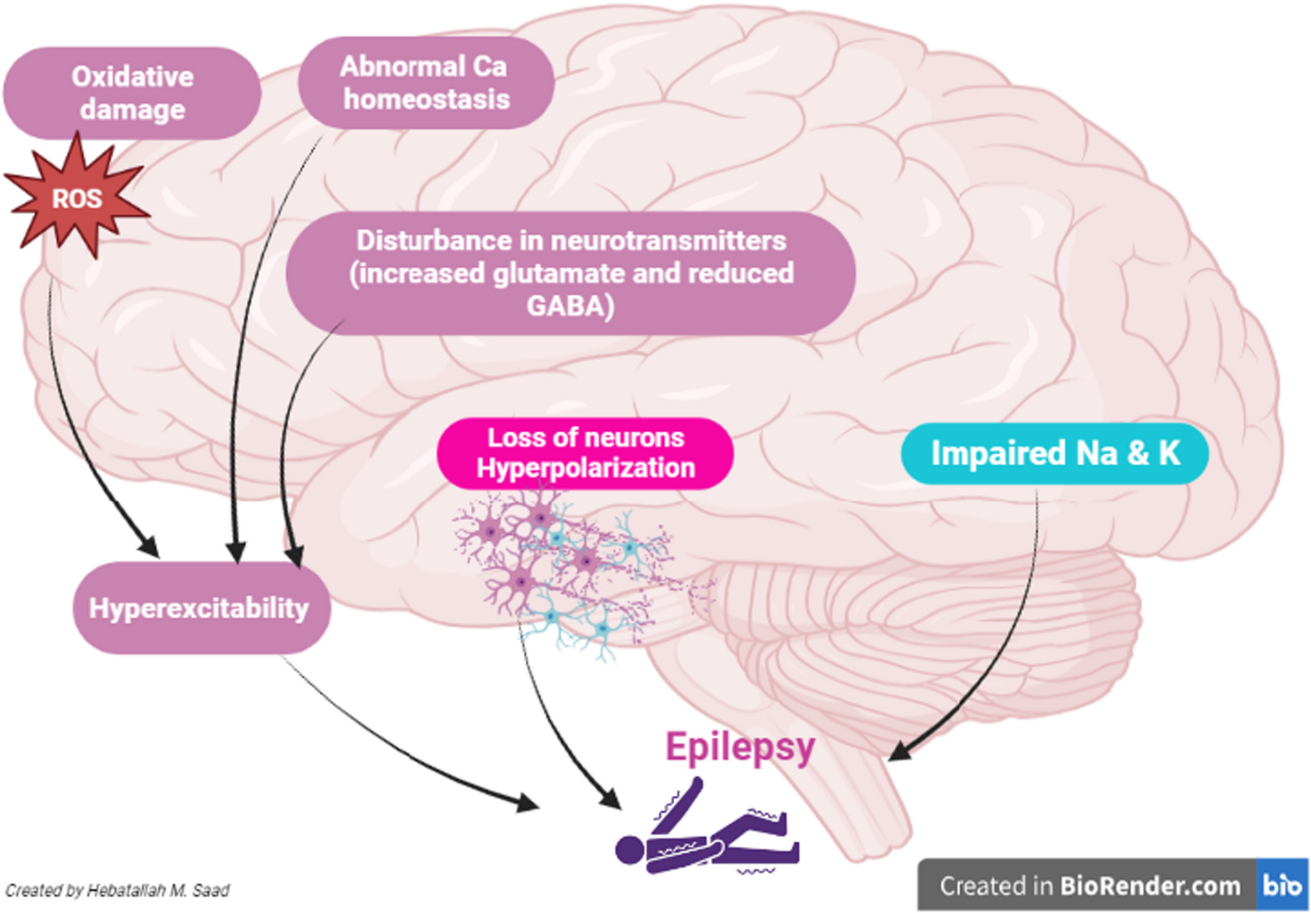
Pathophysiology of epilepsy. Oxidative stress, reduction of inhibitory gamma‐aminobutyric acid (GABA) and increase of excitatory glutamate induce the development and progression of epileptogenesis with the development of epilepsy. Mutation of voltage‐gated Na^+^, Ca^2+^ and K^+^ channels provokes neuronal hyper‐excitability.

The underlying mechanism of epileptic seizure is due to the epileptogenesis process which occurs by imbalance between inhibitory and excitatory neurotransmitters.^14^ Reduction of inhibitory gamma‐aminobutyric acid (GABA) and increase of excitatory glutamate induce the development and progression of epileptogenesis.^15^ The reason for such imbalance is greatly unknown, though mutation of voltage‐gated Na^+^, Ca^2+^ and K^+^ monovalent ion channels provokes neuronal hyper‐excitability and decreases seizure threshold.^16^ Of note, mutation of Na^+^ channel gene SCN8A is linked with the development of epileptogenesis (Figure 1).^17^ The underlying cause of primary epilepsy which is also called cryptogenic epilepsy is unknown.^18^ Nonetheless, secondary epilepsy is caused by different causes including head trauma, brain infection, tumours and neurodegenerative disorders.^19^

Epilepsy is diagnosed by electroencephalogram (EEG), though a normal finding does not exclude epileptic seizure.^20^ Epilepsy is regarded as a controllable disease by anti‐epileptic agents (AEAs) in about 69%.^21^ However, 30%–40% of epileptic patients fail to respond to the conventional AEAs, so‐called refractory epilepsy which may be progressive, increasing the risk for brain structural injury and mortality.^22^ In developing countries, about 90% of epileptic patients are not treated by proper AEA.^23^ Despite the availability of different AEAs of varying mechanisms, the net outcomes were not improved.^23^ The underlying causes for poor clinical outcomes of AEAs are related to different causes including poor safety profile, drug resistance, development of serious adverse effects and high cost mainly second‐generation AEAs.^24^, ^25^ Therefore, adding some FDA‐approved drugs that have an anti‐epileptic activity to the anti‐epileptic regimen is logical. However, this new clinical use could be prohibited in some countries until the drug is approved for the treatment of this other type of disease. Different studies revealed that the anti‐diabetic agent metformin has anti‐seizure activity.^10^, ^26^, ^27^ However, the underlying mechanism of metformin anti‐seizure activity was not fully elucidated. Therefore, the objective of this review was to clarify the mechanistic role of metformin in epilepsy.

## 2 PHARMACOLOGY OF METFORMIN

Metformin is an insulin‐sensitising drug used primarily as a first line in the management of Type 2 diabetes (T2D).^28^, ^29^ Besides, metformin is also used in treating hyperinsulinaemia and hyperandrogenism by improving insulin sensitivity in women with polycystic ovarian syndrome.^28^ Furthermore, preclinical studies confirmed that metformin has antiviral, antibacterial and anti‐tumour activities.^30^

Metformin was discovered in 1920 from a natural product Galegine which was revealed to decrease blood glucose in humans.^31^ Metformin was missed till 1950 when it was introduced in the management of T2D patients.^31^ Metformin belongs to the biguanide group and has a unique complete chemical structure.^32^

Despite the long‐term use of metformin, its mechanism of action is not fully understood.^33^ However, the main mechanism of action of metformin is related to stimulating AMPK which regulates energy balance and body homeostasis. The low dose of metformin does not activate AMPK directly but activates liver kinase beta 1 (LKβ1) which triggers the activation of AMPK.^34^ The positive charge of metformin promotes its accumulation within the mitochondria by about 1000‐folds leading to inhibition of the mitochondrial respiratory chain with augmentation of AMPK.^35^ Metformin is absorbed orally from the small intestine, not metabolized by the liver, does not bind plasma protein, and is excreted unchanged by urine.^36^ Concerning drug–drug interactions between metformin and AEAs, there is no interaction with AEA lacosamide.^37^ In addition, the concomitant administration of metformin with anti‐epileptic topiramate was safe.^38^ Likewise, there are no drug–drug interactions between metformin and valproate.^39^

## 3 METFORMIN AND EPILEPSY

Metformin had been confirmed to inhibit epileptogenesis in pentylenetetrazole (PTZ)‐induced epilepsy in animal models.^26^ It has been illustrated that neuronal expression of AMPK was reduced in animals with acute seizures and chronic epilepsy.^26^ Chronic metformin use shortens the period of brain epileptic seizure activity, and generalized seizure and attenuates post‐ictal depression.^26^ Metformin has a neuroprotective effect against different neurodegenerative disorders. Metformin improves cognitive dysfunction in animal models of epilepsy.^10^ Notably, metformin together with caloric restriction in animal model studies reduces epileptic seizure risk by increasing AMPK and reducing the mechanistic target of rapamycin (mTOR) which is implicated in the induction of epileptogenesis.^27^ A systematic review of the effect of metformin on epilepsy in an animal model study revealed the effectiveness of metformin against the development and progression of epileptic seizure.^40^ Similarly, metformin attenuates pentylenetetrazole (PTZ)‐induced epilepsy in animal models.^41^ Temporal lobe epilepsy represents the most common resistant form of epilepsy but responds to caloric restriction and a ketogenic diet.^42^ An experimental study conducted by Meherabi et al.^42^ demonstrated that metformin was effective in treating temporal lobe epilepsy and status epilepticus in rats. Furthermore, pretreatment with metformin reduced inflammatory cytokines and increased the neuroprotective progranulin and anti‐inflammatory cytokines in rats with experimental temporal lobe epilepsy.^43^ A randomized clinical trial revealed that metformin reduced epileptic seizure frequency in children with tuberous sclerosis.^44^ In addition, metformin is effective against Lafora disease which is a progressive form of myoclonic epilepsy due to a mutation in the EPM2A gene.^45^ A cohort study involved 18 patients with Lafora disease, 8 treated with metformin and 10 untreated showed that metformin was effective in reducing epileptic seizure severity and frequency.^45^

These preclinical and clinical findings indicated metformin could be an effective agent against epileptic seizures mainly the refractory ones.

## 4 THE MECHANISTIC ROLE OF METFORMIN IN EPILEPSY

### 4.1 Metformin mitigates diabetes–epilepsy loop

T2D is an endocrine disease due to the development of insulin resistance (IR) and pancreatic β dysfunction leading to hyperglycaemia and cardiometabolic disorders.^46^, ^47^ Of note, T2D is associated with the development of neurodegenerative diseases. Interestingly, there is a mutual association between T2D and epilepsy, and vice versa.^48^ It has been reported by different studies that T2D patients have a higher tendency to develop epilepsy.^49^, ^50^ Huang et al.^51^ observed that epileptic seizure is more common in patients with diabetic hyperglycaemia as compared with patients with non‐diabetic hyperglycaemia. A prospective comparative study included 41 patients with diabetic hyperglycaemia and 70 patients with non‐diabetic hyperglycaemia showed that seizure severity was correlated with HbA1c.^51^ Besides, epilepsy predisposes to the development of T2D due to increase cortisol release which causes IR and T2D.^52^, ^53^ In addition, psychological stress, social isolation and AEAs‐induced eating disorders predispose to obesity and IR in epileptic patients.^54^, ^55^ These findings highlighted a positive feedback loop between T2D and epilepsy. In this state, treatment of T2D patients by metformin decreases hyperglycaemia and cardiometabolic complications which are comorbid risk factors for the development of epilepsy.^56^, ^57^ Also, metformin attenuates brain IR which is associated with the severity of seizure.^58^, ^59^ Central IR is regarded as a possible connection between T2D and epilepsy as well as neurodegenerative disorders such as Alzheimer's disease (AD).^59^ Moreover, the treatment of epileptic patients with valproic acid predisposes them to the development of IR mainly in obese patients.^60^ A prospective study on 20 epileptic patients treated with valproic acid revealed that only obese patients were predisposed to the progression of IR after 1 year of treatment.^60^ A recent experimental study demonstrated that metformin in combination with a sub‐optimal dose of valproic acid reduces seizure score, improves memory function and attenuates valproic acid‐induced hepatotoxicity and IR.^61^ Of note, first‐generation AEAs such as phenytoin, carbamazepine, valproic acid and phenobarbital are linked with hypercholesterolaemia, metabolic syndrome and vascular risk factors.^62^, ^63^, ^64^ Recently, hypercholesterolaemia and metabolic syndrome are regarded as risk factors for the progression of resistant epilepsy.^65^ Of interest, metformin has been shown to effective in attenuating hypercholesterolaemia and metabolic syndrome according to findings obtained from preclinical and clinical studies.^66^, ^67^ Therefore, metformin in combination with AEAs plays a great role in the management of epilepsy by reducing IR, hyperglycaemia and cardiometabolic disorders which are associated with seizure severity and poor clinical outcomes in epileptic patients. Thus, metformin cuts the feeding loop between T2D and epilepsy.

### 4.2 Metformin and AMPK/mTOR pathway

AMPK and mTOR are highly expressed in the brain and interrelated mutually in the regulation of energy balance and homeostasis.^68^ AMPK which is activated by starvation and metformin activates the catabolic pathway and inhibits the anabolic pathway.^69^ However, mTOR which is activated by high energy, activates the anabolic pathway and inhibits the catabolic pathway.^70^ Notably, mTOR is a cellular molecular sensor serine kinase belonging to the PI3K family that regulates mRNA translation, protein synthesis and cell proliferation.^70^ Stimulating AMPK by metformin triggers inhibition of the mTOR pathway either directly or indirectly. In addition, metformin can inhibit mTOR via the AMPK‐independent pathway.^71^ AMPK has a neuroprotective effect against glucose deprivation and protects astrocytes from apoptosis.^72^ Furthermore, AMPK improves the expression of glucose transporter 1 (GLUT1) which is expressed in astrocytes and regulates central glucose homeostasis.^73^ Deletion or mutation of astrocyte GLUT1 induces seizures in patients with GLUT1 deficiency syndrome.^73^ AMPK enhances glucose uptake and glycolysis of astrocytes by increasing the translocation of membrane GLUT1.^74^ However, the over‐activation of AMPK during brain ischaemia has deleterious effects.^75^ In brain ischaemia, AMPK is activated in the astrocytes due to an increase of nitric oxide (NO) which inhibits mitochondrial respiration and promotes glycolysis.^75^ Moreover, AMPK induces the expression of peroxisome proliferator‐activated receptor gamma co‐activator 1 alpha (PGC1‐α) which improves mitochondrial biogenesis and upregulates sirtuin 1 (SIRT1) and forkhead box o3 (FOXO3) which induces neuroprotection.^76^, ^77^ As well, AMPK inhibits the synthesis of fatty acids and increases their degradation by increasing the expression of malonyl‐CoA which inhibits fatty acid oxidation.^76^ Metformin through the AMPK‐dependent pathway inhibits gluconeogenesis in both astrocytes and neurons leading to an increase in glucose flux and increase glycolysis in astrocytes.^78^ Concerning the anti‐seizure activity of AMPK, it has been shown that AMPK modulates thalamic spike wave seizure in hypoglycaemia‐induced absence seizure. In experimental rats, administration of AMPK agonist metformin potentiates epileptic seizure network activity via activation of postsynaptic GABA_B_ in the thalamocortical neurons.^79^ However, metformin like other AEAs such as tiagabine and vigabatrin can trigger absence epileptic seizure by increasing the availability of GABA which induces stimulation of GABA_B_.^80^ Metformin like other AEAs such as carbamazepine is effective against temporal lobe epilepsy but exacerbates the absence of epileptic seizure.^81^ Absence epileptic seizure is common in children where metformin is rarely used. Therefore, the anti‐seizure effect of metformin seems to be identical to the effect of AEAs which are effective for generalized but not for absence epileptic seizure.

Furthermore, some types of epilepsy are associated with upregulation of the mTOR pathway, and inhibition of this pathway by AMPK activators such as metformin can reduce the frequency and severity of epileptic seizure.^82^ In addition, electroconvulsive shock (ECS) is used in severe depression and can reduce epileptic seizure severity through activation of AMPK and inhibition of mTOR which is involved in epileptogenesis.^82^ Russo et al.^83^ reveal that mTOR inhibitor rapamycin attenuates lipopolysaccharide (LPS)‐induced absence seizure in rats through modulation of neuroinflammation. However, metformin which inhibits the mTOR pathway may exacerbate the absence of seizure.^79^ Therefore, mTOR inhibitors exert different mechanistic pathways against seizure neuro‐activity. Of interest, the ketogenic diet inhibits brain epileptic seizure activity by constraining the expression of mTOR and induces the expression of neuronal AMPK.^84^ It has been shown that the ketogenic diet and its substrates β‐hydroxybutyrate attenuate astrogliosis and mTOR activation in mice with epilepsy.^84^ Yum et al.^85^ observed that β‐hydroxybutyrate increases epileptic seizure threshold and reduces epileptic seizure severity in mice with pilocarpine‐induced seizures. Notoriously, not all mTOR inhibitors are effective in mitigating epileptogenesis.^86^ For example, experimental mTOR inhibitors such as AZD8055 and PF4708671 were shown to be ineffective in mice with epilepsy.^86^ As well, vigabatrin which inhibits mTOR pathway delays but not prevents seizure occurrence in the animal model study.^86^ However, despite these findings, different studies highlighted that the mTOR pathway may exert a protective role against epileptogenesis.^87^, ^88^ It has been shown that microglial mTOR plays a protective role in mitigating neuronal loss and attenuating epileptogenesis in the excitatory injury model of epilepsy.^88^ An experimental study in mice with restrictive deletion of mTOR in microglia revealed that mTOR‐deficient microglia lost their typical proliferative and inflammatory responses to excitatory injury, whereas the proliferation of astrocytes was preserved. In addition, mTOR‐deficient microglia did not effectively engulf injured/dying neurons. More importantly, microglial mTOR‐deficient mice displayed increased neuronal loss and developed more severe spontaneous seizures.^88^ Recent evidence suggests that autophagy impairment is implicated in the epileptogenic mechanisms downstream of mTOR hyperactivation. This holds true for a variety of genetic and acquired epileptic syndromes besides malformations of cortical development which are classically known as mTORopathies. Autophagy suppression is sufficient to induce epilepsy in experimental models, while rescuing autophagy prevents epileptogenesis, improves behavioural alterations and provides neuroprotection in seizure‐induced neuronal damage. The implication of autophagy in epileptogenesis and maturation phenomena related to seizure activity is supported by evidence indicating that autophagy is involved in the molecular mechanisms that are implicated in epilepsy.^89^ In general, mTOR‐dependent autophagy regulates the proliferation and migration of inter‐/neuronal cortical progenitors, synapse development, vesicular release, synaptic plasticity and importantly, synaptic clustering of GABA_A_ receptors and subsequent excitatory/inhibitory balance in the brain. Similar to autophagy, the ubiquitin‐proteasome system is regulated downstream of mTOR, and it is implicated in epileptogenesis. Thus, mTOR‐dependent cell‐clearing systems are now taking centre stage in the field of epilepsy.^89^

**FIGURE 2:**
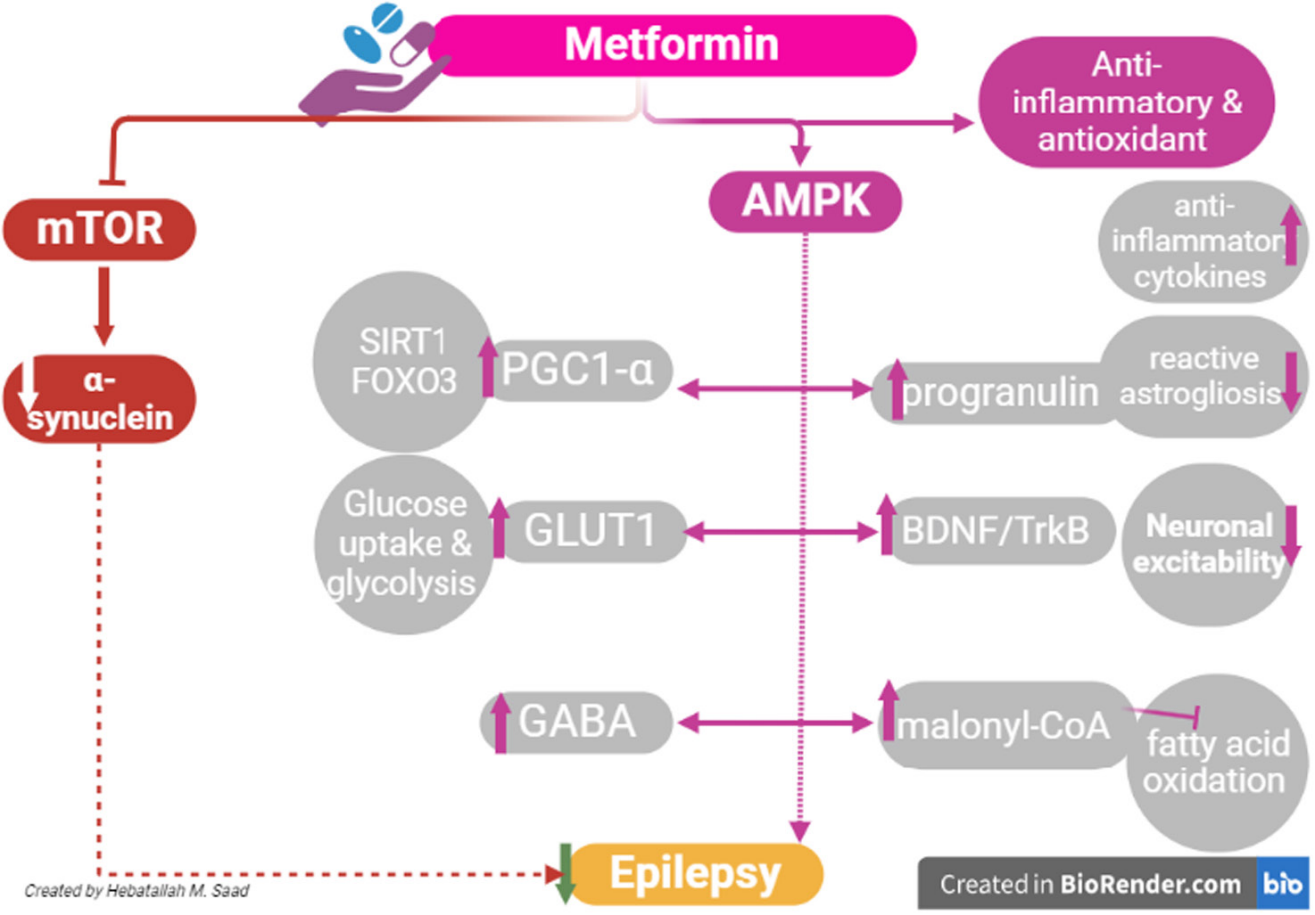
Mechanisms underlying the antiepileptic effect of metformin. Metformin has anti‐seizure activity by activating AMPK signalling and inhibiting the mechanistic target of the rapamycin (mTOR) pathway which are dysregulated in epilepsy. AMPK induces the expression of peroxisome proliferator‐activated receptor gamma co‐activator 1 alpha (PGC1‐α) which improves mitochondrial biogenesis and upregulates sirtuin 1 (SIRT1) and forkhead box o3 (FOXO3) which induces neuroprotection. AMPK enhances glucose uptake and glycolysis of astrocytes by increasing the translocation of membrane glucose transporter 1 (GLUT1). AMPK increases progranulin and brain‐derived neurotrophic factor (BDNF)/tyrosine kinase receptor B (TrkB) and inhibits the synthesis of fatty acids and increases their degradation by increasing the expression of malonyl‐CoA which inhibits fatty acid oxidation.

These findings suggest that metformin has anti‐seizure activity by activating AMPK signalling and inhibiting the mTOR pathway which are dysregulated in epilepsy (high mTOR and low AMPK) (Figure 2).

### 4.3 Brain‐derived neurotrophic factor

Brain‐derived neurotrophic factor (BDNF) is a member of the neurotrophins protein family that is actively involved in neuronal regulation and injury resistance.^90^ BDNF acts on tyrosine kinase receptor B (TrkB) and p75NT receptor (p75NTR).^91^ BDNF is released from peripheral tissues and the CNS mostly the hypothalamus, hippocampus and limbic system.^91^, ^92^ The peripheral action of BDNF is largely related to regulating glucose homeostasis and insulin sensitivity.^93^ Therefore, BDNF is viewed as metabokine due to its outcome on blood lipid and glucose metabolism.^94^ Therefore, BDNF has central and peripheral protective effects.

BDNF serum level is decreased in mice with IR.^95^ Moreover, frequent clinical studies established that BDNF serum levels were reduced in T2D patients compared to controls.^96^, ^97^ In opposition, other studies exhibited that BDNF serum levels were correlated with the level of IR.^94^, ^98^ However, BDNF serum levels were not meaningfully varied in T2D patients compared to the controls.^99^ Of note, BDNF is involved in epileptogenesis by increasing neuronal excitability. Expression of BDNF mRNA is correlated with epileptic seizure activity and epileptogenesis.^100^ A systematic review illustrated that BDNF/TrkB is exaggerated in epilepsy and linked with seizure severity.^101^ Remarkably, acute intra‐cerebral administration of BDNF induces epileptic seizure in mice.^101^ Conversely, chronic infusion of BDNF in mice reduces neuronal excitability by downregulating TrkB and increases the expression of neuroprotective neuropeptide Y (NPY).^101^ Furthermore, BDNF serum level is higher in epileptic patients and correlated with disease severity mainly in temporal lobe epilepsy.^101^ In chronic epilepsy, BDNF is upregulated leading to disruption between inhibitory and excitatory neuronal signalling pathways causing seizures.^102^, ^103^ BDNF increases excitatory glutamate and reduces inhibitory GABA leading to the induction of epileptic seizure.^102^, ^103^ A case–control study on 12 patients with psychogenic non‐epileptic seizure (PNES), 15 patients with an epileptic seizure and 17 healthy controls revealed that BDNF level serum was reduced in patients with epileptic seizure as compared to other patients and healthy controls.^104^ This study had a small sample size which affects the causal relationship between epilepsy and BDNF serum level. Seizure in temporal lobe epilepsy which is a severe and resistant form of epilepsy induces upregulation of BDNF/TrkB.^105^ Deletion of TrkB in animal model study eliminates epileptogenesis while activation of TrkB by oestrogen triggers epileptogenesis in female rats.^105^ Consequently; BDNF/TrkB signalling is intricate in the development and progression of epilepsy. Notoriously, a systematic review illustrated that BDNF serum level in epileptic patients was identical to the general population.^106^ As well, BDNF serum level is reduced in patients with partial epilepsy.^106^ Therefore, there is a strong controversy concerning BDNF serum levels in epilepsy and its subtypes.

On the other hand, BDNF may exert a beneficial effect against the progression of epileptic seizures by enhancing the inhibitory GABAergic neurotransmission.^106^, ^107^ In addition, BDNF reduces neuronal excitability by increasing NPY.^101^ NPY is regarded as an endogenous anti‐seizure via activation of Y2‐Y5 receptors expressed in neurons.^108^ Thus, NPY‐based gene therapy may be a novel AEA for resistance epilepsy. BDNF is reduced in epileptic patients due to stress.^104^ Likewise, AEAs can downregulate BDNF expression leading to a reduction of BDNF serum level in epileptic patients.^109^ Interestingly, TrkB agonists prevent post‐traumatic epilepsy by inhibiting epileptogenesis.^110^ Indeed, BDNF/TrkB role is different according to specific brain regions, it reduces neuronal excitability in the neocortex but augments neuronal excitability in the hippocampus.^110^ Further, continuous administration of BDNF by a bio‐delivery system attenuates generalized epilepsy in rats.^111^ Thus, BDNF/TrkB signalling seems to be beneficial rather than harmful in epilepsy, and increasing BDNF levels in epilepsy could be a compensatory mechanism to prevent epileptic seizure‐induced neuronal injury.^112^

Different studies revealed that metformin improves expression and BDNF levels in experimental cerebral ischaemia in animal models via an AMPK‐dependent pathway.^113^, ^114^ Likewise, metformin improves age‐mediated neurocognitive impairment through the activation of BDNF/TrkB signalling.^115^ Metformin has been shown to enhance neurogenesis and spatial memory formation in adult mice through increased phosphorylation of atypical protein kinase C (aPKC), activation of CREB binding protein (CBP) and activation of BDNF signalling.^115^ Furthermore, metformin has been shown to reduce epileptic seizure in experimental animals through modulation of BDNF/TrkB signalling.^10^ Metformin has shown promising utility in epilepsy management and epileptogenesis modulation by activating BDNF/TrkB signalling.^116^ In the rat pilocarpine model of temporal lobe epilepsy, metformin attenuates epileptic seizure‐induced activation of BDNF/TrkB signalling.^42^ However, other studies indicated that metformin reduced BDNF/TrkB signalling.^10^ In fact, metformin modulates the expression of BDNF which increased following epileptic seizure as a compensatory mechanism. Therefore, metformin via induction of BDNF can reduce seizure progression and severity (Figure 2).

### 4.4 Progranulin

Progranulin is a conserved secreted protein expressed by different cell types in the CNS and peripheral tissues. Progranulin controls cell growth and inflammation, lysosomal function and microglial response.^117^ In the CNS, progranulin is mainly expressed by microglia and induces uptake of synaptophysin by microglia.^118^ Mutation of progranulin is linked with the development of frontotemporal dementia and other neurodegenerative disorders.^119^ It has been shown that progranulin expression is increased in the hippocampus after status epilepticus in mice as a compensatory mechanism.^120^ In addition, progranulin expression is augmented by macrophages and microglia in the hippocampus, cerebral cortex and thalamus within 48 hr, following pilocarpine‐induced status epilepticus.^121^ Besides, CSF progranulin was documented to be increased in epileptic patients following status epilepticus compared to control.^120^ A cohort study on patients with resistance epilepsy (*n* = 56) revealed that CSF progranulin level was increased compared to healthy subjects (*n* = 36).^122^ Of note, metformin activates the expression of neuroprotective and anti‐inflammatory progranulin.^123^ Findings from an experimental study showed that pretreatment with metformin increases progranulin which improves anti‐inflammatory cytokines and reduces reactive astrogliosis.^43^ Deficiency of neuronal progranulin due to mutation promotes complement activation which enhances the engulfment of inhibitory synapses by microglia.^118^ Therefore, increasing progranulin in epilepsy mainly after status epilepticus could be a compensatory mechanism to protect inhibitory synapses from injury by microglia. Augmentation of neuronal progranulin by metformin may explain the anti‐epileptic mechanism of metformin (Figure 2).

### 4.5 α‐synuclein

Synucleins are highly abundant proteins in the CNS that control synaptic vesicle trafficking and neurotransmitter release.^124^ However, the main physiological function of synucleins is not well elucidated.^124^ Synuclein proteins are classified into three types, α, β and γ synucleines. Both α and β synucleins are found in the nerve terminals while γ‐synuclein is present throughout neurons.^125^ α‐synuclein is highly involved in the formation of Lewy bodies a hallmark of Parkinson's disease and other neurodegenerative diseases such as dementia and Alzheimer's disease (AD). The mechanism of α‐synuclein‐induced neurodegeneration is not well understood. However, the formation of neurotoxic α‐synuclein filaments might be the possible mechanism.^126^ It has been shown that epilepsy and neurodegenerative diseases such as AD and PD share a frequent underlying mechanism.^127^ Released α‐synuclein from injured neurons activates astrocytes and microglia leading to neuroinflammation and degeneration of inhibitory neurotransmitters with subsequent induction of epileptogenesis.^127^ Interestingly, α‐synuclein expression is augmented in the hippocampus in rats with PTZ‐induced seizure.^128^ Besides, α‐synuclein expression is higher in epileptic brains as compared to normal brains and correlated with disease severity.^128^ Similarly, pilocarpine‐induced seizure in mice triggers expression of α‐synuclein in the brain within 4 weeks from induction of epilepsy.^129^ In clinical settings, it has been reported that α‐synuclein expression in the brain of patients with temporal lobe epilepsy was increased.^130^ Serum α‐synuclein level is increased in epileptic children correlated with disease severity and cognitive dysfunction.^131^ Of interest, serum α‐synuclein level is correlated with CSF α‐synuclein level and IL‐6.^132^ Remarkably, serum and CSF α‐synuclein levels are augmented in patients with refractory epilepsy.^133^ These findings give a clue that epilepsy is associated with neurodegenerative disorders, and α‐synuclein serum level could be a diagnostic and prognostic biomarker of refractory epilepsy. Therefore, targeting α‐synuclein may reduce epileptogenesis in patients with neurodegenerative disorders and epilepsy.^132^

Numerous studies revealed that metformin can reduce α‐synuclein.^134^, ^135^ Metformin reduces the serum level of phosphorylated‐Ser129 α‐synuclein which is a modified form of α‐synuclein in primary cultured hippocampal neurons through inhibition of the mTOR pathway and modulation of protein phosphatase 2A (PPA2).^135^ Supporting this notion, mTOR inhibitor rapamycin produced similar effects.^135^ PPA2 is regarded as a main tau phosphatase that reduces tau phosphorylation, and induction of PPA2 by metformin may reduce AD neuropathology.^136^ Findings from in vitro and in vivo studies indicated that α‐synuclein inhibits PPA2.^137^ Soluble α‐synuclein activates PPA2, however, over‐expression of α‐synuclein or deposition with the Lewy bodies inhibit PPA2.^137^ A preclinical study demonstrated that administration of metformin 200 mg/kg/day for 7 days in mice with the 1‐methyl‐4‐phenyl‐1,2,3,6‐tetrahydropyridine (MPTP) PD model reduced the expression of brain α‐synuclein through induction of PPA2 and BDNF.^135^ An experimental study on 40 albino rats showed that daily administration of metformin 200 mg/kg IP for 2 weeks attenuates PTZ‐induced epilepsy by reducing α‐synuclein expression in the hippocampus.^128^ These verdicts indicated that metformin may be effective against epilepsy by reducing α‐synuclein and increasing PPA2 (Figure 2).

### 4.6 Neuroinflammation

Neuroinflammation is an immune response of the CNS to exogenous infectious agents or endogenous neurological disorders as in neurodegenerative diseases.^138^ Microglia and astrocytes are involved in the development of neuroinflammation; however, peripheral immune cells that traverse the injured blood–brain barrier (BBB) can contribute to the development of neuroinflammation in chronic inflammatory disorders such as T2D.^139^ Neuroinflammation in the acute phase is protective to eliminate the underlying cause, though chronic neuroinflammation involves progressive neuronal injury, synaptic dysfunction and exacerbation of brain neuropathology.^138^, ^140^ It has been shown that refractory epilepsy is associated with neuroinflammation and activation of the mTOR pathway which is involved in epileptogenesis.^141^ Parson et al.^142^ illustrated that neuroinflammation together with induced oxidative stress by inflammatory reactions increases neuronal excitability. Oxidative stress due to neuroinflammation induces aberrant neuronal signalling via activation of inflammatory signalling pathways such as nuclear factor kappa B (NF‐κB) and mitogen‐activated protein kinase (MAPK) which induce neuroinflammation progression.^143^ These changes enhanced the release of pro‐inflammatory cytokines which induce oxidative stress and neuronal excitability.^144^ Neuroinflammation is highly intricated in the epileptogenic cortical area in humans and animals.^144^ Biomarkers of neuroinflammation such as IL‐1, toll‐like 4 receptor and transforming growth factor beta (TGF‐β1) could be a potential biomarker for epilepsy resistance.^144^ Therefore, anti‐inflammatory and antioxidant agents could be effective in resistance to epilepsy.^145^ Of note, metformin has anti‐inflammatory and antioxidant effects,^146^ therefore it could be effective in attenuating neuroinflammation and associated epilepsy. Yimer et al.^40^ observed that metformin alleviates symptoms of epileptic seizure and modifies different cellular and molecular changes that affect the natural history of the disease through its anti‐inflammatory and antioxidant effects. Numerous studies have shown the potential role of metformin to modify these cellular and molecular alterations in an animal model of various neurological disorders including epileptic seizure by modulating the expression and release of proinflammatory cytokines as well as markers of oxidative stress.^147^, ^148^ Metformin is also capable of deterring molecular alterations including oxidative stress which is a peculiar factor that plays an enormous role in the initiation and progression of epileptogenesis.^149^ Mitochondrial dysfunction and abnormal gene expression of oxidative markers involved in scavenging reactive oxygen and nitrogen species have resulted in a profound increment of free radicals and impairment of brain mitochondrial oxygen consumption. All these are suggested to contribute to epileptogenesis.^149^ Interestingly, metformin showed antioxidant activities through attenuation of oxidative free radicals including lipid peroxidation and advanced glycation end‐products and improved the antioxidant defence system including superoxide dismutase, catalase and glutathione levels in addition to its effects on seizure outcomes.^150^ Similarly, various studies showed the ability of metformin to mitigate the release and production of endogenous proinflammatory mediators including *Phospho*‐*IkBα*, TNF*α*, IL‐1*β*, IL‐6 and vascular endothelial growth factor (VEGF) during epileptogenesis in animal model studies.^151^ Furthermore, metformin also exhibited a substantial reduction of cellular apoptosis induced by PTZ by modifying the expression of caspase‐3 and ‐9 in metformin‐pretreated epileptic mice.^152^ Thus, in virtue of its anti‐inflammatory and antioxidant effects, metformin can mitigate epileptogenesis and the development of epilepsy.

It has been shown that metformin decreases neuroinflammation and enhances cognitive function following traumatic brain injury in mice.^153^ Likewise, metformin‐loaded phosphatidyl nanoliposomes promote memory function and decrease neuroinflammation in streptozotocin‐induced AD in mice.^154^ Long‐term use of metformin reduces neuroinflammation and prevents the development of AD in patients with T2D.

These observations proposed that metformin could be effective against epileptogenesis by attenuating neuroinflammation.

In sum, these verdicts proposed that metformin attenuates epileptogenesis and epilepsy by indirect mechanisms mainly by modulating inflammatory and oxidative stress disorders, BDNF, α‐synuclein, mTOR pathway and neuroinflammation. However, direct evidence for the effect of metformin on the epileptogenesis process and epilepsy is limited. An experimental study observed that metformin improves GABAergic neurotransmission in diabetic rats by regulating brain metabolism and insulin signalling in diabetic rats.^155^ Recent studies suggest that metformin appears not only to regulate synaptic transmission or plasticity in pathological conditions including epilepsy but also to regulate the balance of excitation and inhibition balance in neural networks.^156^ Metformin can promote the membrane insertion of GABA_A_ receptor and enhance the inhibitory synaptic neurotransmitter function and micro‐inhibitory postsynaptic currents in cultured rat hippocampal neurons by activating AMPK‐FOXO3A signalling pathway and increasing the expression of GABA_A_ receptor‐associated protein.^157^ In a rat model of diabetic epilepsy, metformin corrected the abnormal level of glutamate and GABA values in the hippocampus.^158^ In an open‐label study, increased corticospinal inhibition mediated by the GABA_A_ and GABA_B_ mechanisms was observed by transcranial magnetic stimulation in patients with metformin treatment, suggesting the potential of metformin in modifying GABA‐mediated inhibition.^159^ Glutamate excitatory toxicity in nutrient‐deficient cells was mitigated after metformin treatment, mediated partly by the downregulation of AMPK and subsequent reduction in autophagy. Similarly, metformin directly inhibits glutamate‐induced neuronal excitotoxicity by regulating autophagy and MAPK phosphorylation.^160^ In the LPS‐induced depression mouse model, metformin administration reduced presynaptic glutamate release and decreased the miniature excitatory postsynaptic currents (mEPSCs) frequency of hippocampal pyramidal neurons. Metformin treatment restored excitatory synaptic activity in hippocampal sections to normal levels and rescued exaggerated metabolic glutamate receptor‐dependent long‐term depression of synaptic transmission in mice model.^161^ Thus, metformin may affect glutamatergic and GABAergic synapses by directly regulating the number of neurotransmitters released and changing the expression level of receptors on the postsynaptic membrane.

Taken together, metformin has anti‐seizure and anti‐epileptic activities as it attenuates the progression of acute epileptic seizure by inhibiting epileptogenesis in different animal model studies, and attenuates the progression and the development of epilepsy in human studies.

## 5 CONCLUSIONS

Epilepsy is a chronic neurological disorder due to hypersynchronous neuronal discharge from a definite brain area leading to recurrent seizures. The fundamental mechanism of epileptic seizure is due to the development of epileptogenesis which happens by an imbalance between inhibitory and excitatory neurotransmitters. Epilepsy is observed as a manageable disease by AEAs in about 69%; however, 30%–40% of epileptic patients do not respond to conventional AEAs. Consequently, adding some FDA‐approved drugs that have an anti‐seizure activity to the anti‐epileptic regimen is recommended. Noteworthy, the anti‐diabetic agent metformin has anti‐seizure activity. However, the original anti‐seizure activity mechanism of metformin was not completely clarified. Hence, metformin in combination with AEAs may play a pronounced role in the management of epilepsy by reducing IR, hyperglycaemia and cardiometabolic disorders which are associated with seizure severity and poor clinical outcomes in epileptic patients. Consequently, metformin can cut the feeding loop between T2D and epilepsy. These verdicts propose that metformin has anti‐seizure activity by activating AMPK signalling and inhibiting mTOR pathways which are dysregulated in epilepsy. In addition, metformin advances the expression of BDNF which has a neuroprotective effect. Thus, metformin via induction of BDNF can decrease seizure progression and severity. Consequently, increasing neuronal progranulin by metformin may explain the anti‐epileptic mechanism of metformin. Moreover, metformin decreases α‐synuclein and increases PPA2 with modulation of neuroinflammation. Collectively, metformin could be an adjuvant treatment with AEAs in the management of intractable epilepsy. Preclinical and clinical studies are justified in this respect.

## AUTHOR CONTRIBUTIONS

**Saud A. Alnaaim:** Writing – review and editing (equal). **Hayder M. Al‐kuraishy:** Conceptualization (equal); writing – original draft (equal). **Ali I. Al‐Gareeb:** Writing – review and editing (equal). **Naif H. Ali:** Conceptualization (equal); data curation (equal); writing – review and editing (equal). **Athanasios Alexiou:** Resources (equal); validation (equal); visualization (equal). **Marios Papadakis:** Supervision (equal); writing – original draft (equal). **Hebatallah M. Saad:** Writing – original draft (equal); writing – review and editing (equal). **Gaber El‐Saber Batiha:** Supervision (equal); writing – original draft (equal). Open Access funding enabled and organized by Projekt DEAL.

## FUNDING INFORMATION

This work was supported by the University of Witten‐Herdecke Germany.

## CONFLICT OF INTEREST STATEMENT

The authors declare no conflict of interest.

## Data Availability

Data sharing is not applicable to this article as no new data were created or analysed in this study.
